# The Influencing Factors of “Post-African Swine Fever” Pig Farm Biosecurity: Evidence from Sichuan Province, China

**DOI:** 10.3390/ani13193053

**Published:** 2023-09-28

**Authors:** Huan Wang, Meijun Chen, Ziyao Guo, Yangyang Shen, Yufan Chen, Ting Luo, Yuying Liu, Jianqiang Li, Fang Wang, Jiangjun Wan

**Affiliations:** 1 College of Management, Sichuan Agricultural University, Chengdu 611130, China; wanghuan@sicau.edu.cn (H.W.); 2020209057@stu.sicau.edu.cn (M.C.); shenyangyang@stu.sicau.edu.cn (Y.S.); 202003489@stu.sicau.edu.cn (Y.C.); 18224022340@163.com (T.L.); yuying.liu@sicau.edu.cn (Y.L.); ljq9801@126.com (J.L.); wangfangscnd@sicau.edu.cn (F.W.); 2 College of Architecture and Urban-Rural Planning, Sichuan Agricultural University, Chengdu 611830, China

**Keywords:** pig farmers, biosecurity, African swine fever, influencing factors

## Abstract

**Simple Summary:**

African swine fever (ASF) has a significant impact on the pig industry, leading to drastic fluctuations in the supply and price of live pigs on the market, leading to substantial economic losses for farmers. As China enters the ‘post-ASF’ era of regular epidemic prevention and control, the behavior of farmers becomes crucial for effectively preventing and controlling major animal epidemics. To analyze the current situation and factors influencing farmers’ epidemic prevention and control behaviors, survey data were empirically analyzed. The findings indicate that the overall level of biosecurity in pig farms is low. Additionally, factors such as technical training, farm size, income share, production organization, and government inspection significantly influence farmers’ adoption of biosecurity measures.

**Abstract:**

Effective biosecurity measures are crucial in controlling and preventing major pig diseases, ultimately ensuring farm income and social stability. This study extracted data from 205 farmer surveys in Sichuan Province, China, to construct a biosecurity index system for pig farms. The biosecurity levels of pig farms were evaluated using a projection pursuit method to identify weak areas. The Tobit model was then utilized to determine the factors that influenced the biosecurity levels. The results indicated that the overall biosecurity levels of the pig farms were low. The study found that the average biosecurity score among farms was 0.61, with a minimum score of 0.37 and a maximum score of 0.89 (on a scale of 0 to 1). These results suggest that there are significant differences in biosecurity levels among the farms. The study also found that the scores for first-level indicators related to breeding environment management, as well as second-level indicators related to personnel management and awareness of African swine fever prevention and control, were significantly lower than scores for other indicators in the farmers’ biosecurity systems. This study investigated the factors influencing biosecurity on farms and found that technical training, farm size, income share, production organization, and government inspections had a significant impact on the level of biosecurity implemented. This study emphasizes the significance of biosecurity in enhancing pig farm biosecurity and its role in improving farm resilience to major animal diseases like African swine fever. It also provides valuable insights for policymakers to make informed decisions regarding related policies.

## 1. Introduction

African swine fever (ASF), which is a highly contagious, hemorrhagic disease caused by the African swine fever virus (ASFV), can cause substantial morbidity in pig herds, with case fatality approaching 100% [1,2], and human behaviors play an important role in spreading this pig disease across borders. According to the information of The World Organisation for Animal Health (OIE-WAHIS), between December 2021 and January 2022, ASF has been presented in five different world regions in 33 countries, affecting more than 1,000,000 pigs and more than 29,000 wild boars, with more than 1,600,000 animal losses. China accounts for more than half of the world’s pork production and consumption, so volatility in the Chinese and world pig markets has increased since the start of the Chinese ASF epidemic was detected in Liaoning province in August 2018 [3,4]. The Chinese government has since implemented a series of measures to control and cut off the virus transmission chain, including sampling and monitoring on farms, promoting regional prevention and control of major animal diseases, strict quarantine supervision and mandatory culling. Over the course of almost two years, China has made significant improvements in biosecurity protection and regional epidemic prevention capacity. In May 2020, China announced that they had effectively controlled ASF, resulting in no further large-scale or regional outbreaks as seen in previous years. Additionally, pig production capacity has been recovering and market prices have been stabilizing. It indicated that China had entered a “post-African swine fever” era of regular epidemic prevention and control [5]. Farmers have taken on the primary responsibility for preventing and controlling major animal epidemics rather than the government. Their biosecurity prevention and control practices directly impact the effectiveness of government policies implemented to prevent and control such epidemics.

Biosecurity measures can range from broad national policy implementation requirements to narrow management practices at the individual production unit level [6,7]. On the macro level, the Chinese government has prompted pig farmers to support the pig industry, improve biosecurity, and promote stable pig industry development. On the micro level, farmers have the responsibility of preventing and controlling diseases by implementing measures such as cleansing, disinfection, and bio-management on the farm. These measures aim to minimize the risk of pathogenic spread on the farm (bio-exclusion), as well as outward transmission (bio-contamination) and upward transmission through the market chain [8,9,10,11]. Farmers have contributed to the long-term misconception that vaccines are important, but biosecurity is not [3,12,13]. As a result, even in the post-ASF era, most pig farmers do not take disinfection and isolation seriously [14]. There are also limitations associated with the segregation of pigs from insects and rodents [15]. The scale, mode, and management style of Chinese pig farms vary significantly, leading to poor epidemic prevention awareness and inadequate facilities. Additionally, emergency critical response time is low across the board. So, ASF is still presenting in a dispersed and point-like spread pattern in the “post-ASF” era [16]. Given the absence of safe and effective vaccines or antiviral drugs, enhancing biosecurity measures for farmers emerges as the most effective protective measure to prevent and manage disease outbreaks on farms [17,18]. There are behavioral shortcomings in China’s pig farm biosecurity systems [19]. There is currently a noticeable polarization in biosecurity management, with smaller family farms being less able to avoid epidemics [20] due to their weak or nonexistent implementation of epidemic prevention and control measures.

Previous studies on biosecurity measures have commonly utilized the Biocheck. Ugent™ scoring system to assess the biosecurity levels of specific farms by interviewing farmers about their biosecurity practices [21,22]. These articles refer to the external and internal biosecurity aspects related to Purchase and Breeding, Transportation and Carcass Removal, Feed and Water, Visitors and Employees, Pest Control and Other Animals, and Farrowing Management in Biocheck. UGent. Referring to the existing research and combining with the actual pig production in China and the technical guide for disease prevention and control in pig farms, this paper establishes a biosafety scoring system consisting of five components: breeding environment management, breeding process management, personnel and vehicle management, decontamination and waste treatment, and biosafety awareness. Scoring is performed using the projection pursuit model to ensure the reliability of assessment results when biosecurity level assessments were conducted.

Despite the importance of farm biosecurity, there has been a dearth of systematic and comprehensive analysis on the root causes affecting its level. An early study reviewed the factors influencing pig farming biosecurity [23]. The occurrence of African swine fever (ASF) outbreaks had a negative impact on the confidence of smallholder farmers in the effectiveness of biosecurity measures, resulting in lower levels of biosecurity on their farms [24]. However, these studies did not offer a clear explanation for this observation. Therefore, in order to further examine the factors that impact farm biosecurity, in this paper, the Tobit model is used to determine the factors that affect the level of biosecurity.

The study aims to answer three main questions: First, how can the biosecurity level of farmers be calculated scientifically? Second, where are the weak links in biosecurity for farmers? Third, what are the root causes affecting the biosecurity level of farmers? Compared with previous studies, the main innovations of this study are as follows: (i) the micro-survey data of Sichuan Province in 2020 were used to construct a complete biosecurity behavior index system, and the projection tracking model was used to calculate the biosecurity scores of pig farmers and the weak links of the biosecurity system of pig farmers were explored. (ii) The effects of farmers’ basic characteristics, production and management characteristics and breeding environment characteristics on the biosecurity level of pig farmers were analyzed, and the effects of epidemic awareness characteristics were also discussed. (iii) We explore ways to improve the biosecurity level of pig farmers under the influence of the epidemic and provide theoretical and empirical evidence for the construction of a biosecurity system for the prevention and control of pig diseases.

## 2. Theoretical Analysis and Hypotheses

The transmission of ASF is dependent on three critical elements: infection source, infection route, and animal susceptibility. Contact with infected pigs and products derived from them are important sources of ASF virus transmission on farms, including exposure to contaminants and contaminated environments [25,26]. Improving biosecurity is the most fundamental and cost-effective response to prevent the outbreak of epidemics and limit their spread within farm areas. However, the implementation of biosecurity practices can be limited by factors such as cost, cultural traditions, social factors, and the quality of veterinary services, which can affect family livelihoods [27]. Farmers can choose to withdraw from breeding or implement appropriate biosecurity measures when faced with the potential threat of disease, depending on their endowment. However, withdrawing from breeding results in a significant loss of dedicated assets, while implementing biosecurity measures leads to increased prevention and control costs. Improving biosecurity not only stabilizes farming income but also reduces disease treatment costs and the chances of pig morbidity and losses. Improving biosecurity has several benefits, including providing quality and safe pig products, enhancing the ecological environment both on and off the farm, reducing pathogen transmission, and creating a safe living environment for neighboring farmers and communities. By joining a production organization, the resulting positive externalities can be internalized. This is because such organizations offer uniform services that can further reduce the costs of biosecurity prevention and control. For larger-scale biosecurity systems, government intervention is required, which is often too expensive for individual farmers or cooperative production organizations. Information sharing by pig farmers, mandatory risk assessments by veterinary services, and domestic and wild pig disease surveillance can prevent outbreaks such as ASF [28]. Based on these considerations, this paper drew on relevant research findings to identify the four main biosecurity influencing factors [29,30]: personal characteristics, production and operation characteristics, breeding environment characteristics, and epidemic knowledge characteristics.

Personal characteristics comprise gender, production field experience, and the influences on the farmers’ knowledge acquisition, attitudes, and practices toward biosecurity [31]. Generally, female farmers play a key role in pig husbandry and disease control [32]. When farm decision makers are older, they tend to limit themselves to simple, labor-saving tasks and are more conservative in their thinking, which means they are more reluctant to improve their biosecurity [12,33].

Farming experience, farming habits, and education level have also been found to influence biosecurity practices. Farmers with less education may rely on their established habits and practical experience, making them more hesitant to adopt unfamiliar biosecurity practices. Conversely, farmers with higher levels of education tend to be more open to implementing farm hygiene practices to promote better biosecurity measures [34]. Farm decision makers who receive training in biosecurity techniques demonstrate a heightened awareness of the importance of biosecurity. Additionally, those who are familiar with the appropriate techniques are more mindful of the need for adequate biosecurity measures and are more willing to improve their biosecurity practices [8,11]. Therefore, the following hypothesis is proposed.

**H1:**  *Farmer characteristics affect farm biosecurity. Females are more likely to improve biosecurity levels than males, education and technical training have a positive effect, and age has a negative impact.*

In terms of production and operational characteristics, farmers with more years of breeding experience tend to have a better understanding of the significance of biosecurity. However, their reliance on their own breeding experience instead of improving biosecurity measures may lead to variable outcomes, making the influence of years of farming inconsistent. Farm breeding scale, income from breeding as a proportion of total household income, and social responsibility can also moderate ASF response and control behaviors, primarily because of costs and the possible loss of profit [17]. As the size of the farm increases, so does the number of pig-specific assets, leading to a greater sense of risk aversion among farmers. Considering economies of scale, large farms are also likely to be more profitable and therefore, higher investment in biosecurity becomes feasible [35]. Farmers who hold higher social positions may have greater access to and be more willing to adopt biosecurity measures. This could be attributed to their status as public figures, which may influence their sense of moral and social responsibility [17]. As the proportion of farming income increases, households become more reliant on this source of income and are, therefore, more willing to invest in improving biosecurity measures [36]. Therefore, the following hypothesis is proposed.

**H2:**  *Production and operations characteristics influence farm biosecurity levels. In particular, farm size, social position, and farm income percentage all have a positive effect, while the impact of the number of farming years is uncertain.*

Regarding breeding environment characteristics, farms with a higher level of specialization tend to have a greater awareness of disease control [37]. Farmers who are part of a production organization have access to a wider range of resources, including knowledge, technology, capital, and information, which allows them to improve their biosecurity measures. On the other hand, individual farmers may have less financial resources to allocate towards disease prevention and control. Farmers who take out farm insurance are more likely to improve their biosecurity as they have purchased farm insurance to reduce possible epidemic losses [38]. Government involvement in monitoring farm biosecurity measures puts greater regulatory pressure on farmers [12,39]; therefore, the more frequent the on-site government inspections, the higher the risk of being penalized for not complying with biosecurity control requirements. Consequently, the more stringent the government inspections, the greater the likelihood that farmers will implement the required biosecurity measures. Therefore, the following hypothesis is proposed.

**H3:**  *Farming environment characteristics influence farm biosecurity, with production organization, farming insurance, and government inspections having positive impacts.*

Biosecurity knowledge plays a crucial role in shaping farmers’ attitudes and behaviors toward implementing biosecurity measures. Farmers who possess knowledge about biosecurity and diseases are more likely to implement biosecurity measures on their farms [31]. For epidemic perception characteristics, timely reporting and accurate responses to primary ASF cases depend on the familiarity of the pig farmers with the clinical signs of ASF [40]. The more knowledgeable farmers are about the ASF symptom presentation and the current situation, the more likely they would be to avoid an outbreak and the more willing they would be to improve their biosecurity. The lack of attention given to ASF by pig farmers is often related to a lack of awareness of the threats associated with ASF and an unwillingness to bear the costs of changing their traditional animal farming systems [25]. Increased awareness among farmers about the potential losses resulting from culling in the event of an outbreak on their farms would likely lead to an improvement in their biosecurity practices. Reporting morbidity in a timely manner is a crucial component of the biosecurity system. Therefore, farmers who are willing to report significant or suspected cases of animal disease in and around their farms are demonstrating their support for the establishment of biosecurity barriers. Therefore, the following hypothesis is proposed.

**H4:**  *Epidemic awareness characteristics affect a farmer’s biosecurity, with an awareness of ASF symptoms and the mandatory culling policy resulting in a greater willingness to report the epidemic and positive effects on the implementation of biosecurity measures*.

**Figure 1:**
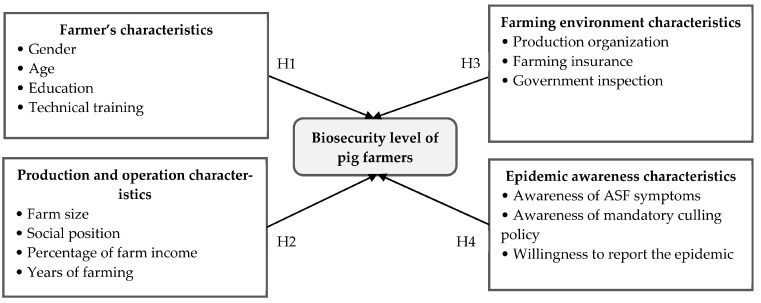
Conceptual framework.

Figure 1 shows the conceptual framework of this study.

## 3. Materials and Methodology

### 3.1. Study Area

**Figure 2:**
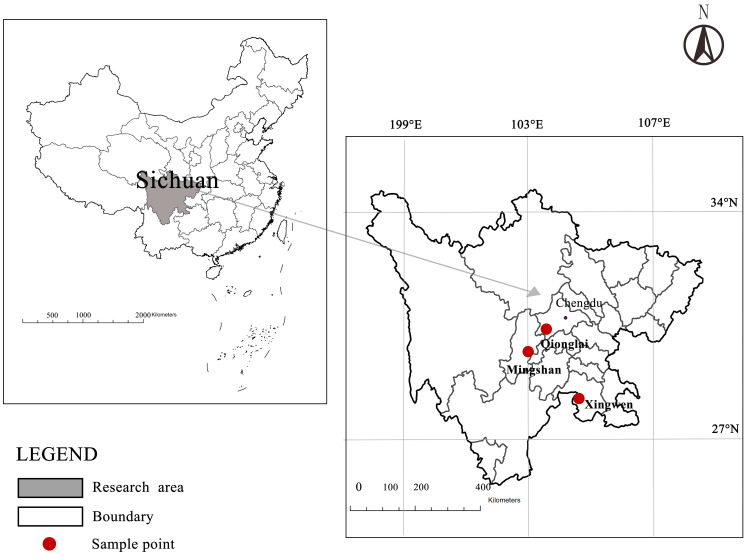
Location of the study area.

To gain a comprehensive understanding of pig farmers’ biosecurity practices in the “post-ASF” era, this article conducted a field survey on the biosecurity of pig farmers. Sichuan Province is located in the southwestern region of China and features an area with high elevation in the East and low elevation in the West. Due to its high ranking in pig production and consumption throughout the year, the pig industry in Sichuan is a representative example of China’s pig industry. The study selected three prefecture-level cities in Sichuan Province based on the number of live pigs sold in 2020. These cities were chosen from the top 30%, top 60%, and bottom 30% of live pig sales, respectively. Each selected city represented a different production level. To ensure sample quality, the research samples initially selected three cities—Chengdu on the Chengdu Plain, Yaan in western Sichuan, and Yibin in southern Sichuan (See Figure 2).

### 3.2. Data Sources

To ensure the reliability of the data, investigators conducted a pre-survey where they interviewed each farmer individually before the formal investigation began in July 2021. The purpose of the pretest survey was to identify any missing or improperly phrased questions, as well as any other potential issues with the questionnaire. Each interview lasted approximately 1 h and covered topics such as the farmers’ decision-making process, characteristics of their household and farm production operations, the biosecurity measures in place on pig farms, and the farmers’ awareness and utilization of epidemic prevention and control techniques.

In the formal investigation, a random stratified sampling method was employed in this study, which considered differences in the volume of pig slaughter to ensure representativeness. Two to three sample villages, one from each prefecture-level city, were randomly selected. Finally, about 30 pig farmers were randomly selected from the provided list of pig farmers as the interviewees in each sample village. Overall, a total of 208 questionnaires were returned, and 205 valid samples were obtained, excluding those with missing or abnormal data. The basic information for the interviewed farmers is shown in Table 1.

Table 1 indicates that the decision makers were mainly male, accounting for 76.10% of the total sample, and 78.05% of the decision makers were older than 45 years old; therefore, male, middle-aged, and older people were the main pig producers. The basic characteristics of the interviewed farmers were consistent with the current state of pig farming in China. The education of the decision makers was relatively low, with only 40.98% having a junior high school education. Members of 152 farming households held social positions, 34.63% had annual pig income that accounted for more than 75% of their total income, and 28.29% had a breeding scale between 100 and 499 pigs, indicating that while the breeding industry was their main income source, the farms were small or medium-sized.

### 3.3. Research Methods and Models

#### 3.3.1. Construction of Biosecurity Index

Theory and related research have demonstrated that basic biosecurity measures include factors such as location and layout, production processes, feed types, daily disinfection, the treatment of dead pigs, management strategies, and infrastructure construction rules and regulations [41,42,43,44,45,46,47]. Based on the related research, the biosecurity level in this study was assessed on five aspects: (1) Breeding environment management, which comprised both offsite and on-site environmental controls, such as scientific site selection, optimized layout, reasonable zoning, regular rodent control, mosquito control, fly control, and the prohibition of unrelated animals; (2) breeding process management, which comprised inputs such as feeding, and slaughter management, formal source channels for pig breeds, feed, medicines and other inputs, appropriate storage to meet the requirements, standardized and orderly breeding operations, breeding file records, and live pig supervision; (3) personnel and vehicle management that met the requirements and complete admission procedures; (4) decontamination and waste treatment, such as cleaning, disinfection, waste treatment, cleaning and disinfection equipment, the timely disinfection of foreign and contaminated objects, and the harmless treatment of aquaculture waste; and (5) biosecurity awareness, such as ASF prevention and biosecurity management awareness regarding ASF and related biosecurity knowledge, epidemic handling procedures, and the importance of improving biosecurity. A biosecurity index system for pig farms was constructed based on the available data. The system comprises five first-level indicators, 11 second-level indicators, and 52 third-level indicators, which are outlined in detail in Table 2.

#### 3.3.2. Calculation of Biosecurity Level

This study utilized the projection pursuit model to weigh the evaluation indexes for the biosecurity level of five target layers of pig farms, which included breeding environment management, breeding process management, personnel and vehicle management, decontamination and waste treatment, and biosecurity awareness. The model calculated the biosecurity level. The evaluation index system constructed in this study is comprehensive, complex, and high-dimensional, which renders traditional evaluation methods like the entropy method, principal component analysis, and analytic hierarchy process inapplicable. The projection pursuit model, a mathematical, statistical model proposed by Kruskal in 1972 for nonlinear and informal high-dimensional data, was used instead to make the weight determination of complex data robust and objectively reasonable. By projecting high-dimensional data into low-dimensional subspaces, the projection pursuit model can match optimal values [48,49], the basic steps for which were as follows.

Indicator standardization: The index system sample set was $\left\{\left.x^{*}(i,j)\right|i=1,2,\cdots ,n;j=1,2,\cdots ,p\right\}$, where $x^{*}(i,j)$ was the jth index value for the ith sample, n was the number of samples, and p was the number of indicators. To eliminate any dimensional influences, Formula (1) was used to normalize the data for each indicator.

(1) $$\left\{\begin{array}{c} x^{*}(i,j)=\frac{x^{*}(i,j)-xmin(j)}{xmax(j)-xmin(j)}\;\;\;\;\;\;For\;positive\;indicators\\ x^{*}(i,j)=\frac{xmax(j)-x^{*}(i,j)}{xmax(j)-xmin(j)}\;\;\;\;\;For\;negative\;indicators \end{array}\right.$$

Construction of the projection objective function Q(a): The p-dimensional data $\left\{\left.x^{*}(i,j)\right|i=1,2,\cdots ,n;j=1,2,\cdots ,p\right\}$ was synthesized into a one-dimensional projection value z(i). With $a=\left\{a(1),a(2),a(3),\cdots ,a(p)\right\}$ being the projection direction,

(2) $$z(i)=\sum_{j=1}^{p}a(j)x(i,j)\;\;\;\;(i=1,2,\cdots ,n)$$

(3) $$Q(a)=S_{z}D_{z}$$

where $S_{z}$ was the standard deviation for the projected value z(i), and $D_{z}$ was the local density of the projected value z(i)

(4) $$S_{z}=\sqrt{\frac{\sum_{i=1}^{n}{\left[z(i)-E(z)\right]}^{2}}{n-1}}$$

(5) $$D_{z}=\sum_{i=1}^{n}\sum_{j=1}^{p}\left[R-r(i,j)\right]u\left[R-r(i,j)\right]$$

where E(z) was the average value for the sequence $\left\{\left.z(i)\right|i=1,2,\cdots ,n\right\}$, R was the window radius for the local density, with the value generally being 0.1, $S_{z}$, r(i,j) was the distance between the samples, and u(t) was a unit step function; when t ≥ 0, the value was 1 and otherwise was 0. 

Optimization of the objective projection function: when the set for each index value was determined, to find the best projection direction, the projection objective function Q(a) changed with the projection direction a. The formula for solving the optimal projection direction was as follows:

(6) $$Max:Q(a)=S_{z}D_{z}$$

(7) $$s.\;t:\sum_{j=1}^{p}a^{2}(j)=1$$

Classification and evaluation: By substituting the optimal projection direction $a^{*}$ into Formula (2), the projection value $z^{*}(i)$ for each sample point was calculated. By arranging $z^{*}(i)$ from large to small, the ranking of samples can be obtained.

#### 3.3.3. Analysis of Influence Factors

To better understand the underlying reasons behind the farmers’ biosecurity scores, it is necessary to analyze the factors that influence their biosecurity levels. While the biosecurity scores only indicate the ability to resist the risk of epidemic diseases, they do not provide insight into the reasons behind the scores. To address this issue, we employed the widely used Tobit model, as the farmer’s biosecurity score is a locally explained variable between 0 and 1. The Tobit model was used to analyze the factors that affect the farmer’s biosecurity level. The model was constructed as follows:

(8) $$y=\beta_{0}+\sum_{i=1}^{n}\beta_{i}x_{i}+u_{i}$$

(9) $$u_{i}\sim (0,\sigma^{2})$$

where y was the explained variable that indicated the biosecurity level of the farmer, $x_{i}$ was the explanatory variable, *i* was the *i* farmer’s endowment, external environmental characteristics, and other factors, *n* was the number of influencing factors, $\beta_{i}$ was the coefficients to be estimated, and $u_{i}$ was a random disturbance term.

### 3.4. Selected Variables

(1) Explained variable: based on the biosecurity index system, the explained variable is the pig farmer’s biosecurity level, which is calculated by using the projection pursuit model.

(2) Explanatory variables: based on the aforementioned theoretical analysis, the explanatory variables that impact the biosecurity of the pig farmers were classified into four aspects: basic personal characteristics, production and operation characteristics, breeding environment characteristics, and epidemic knowledge characteristics. The definitions and descriptive statistics for the explained and explanatory variables are shown in Table 3.

## 4. Results

### 4.1. Analysis of Farmers’ Biosecurity Level

**Figure 3:**
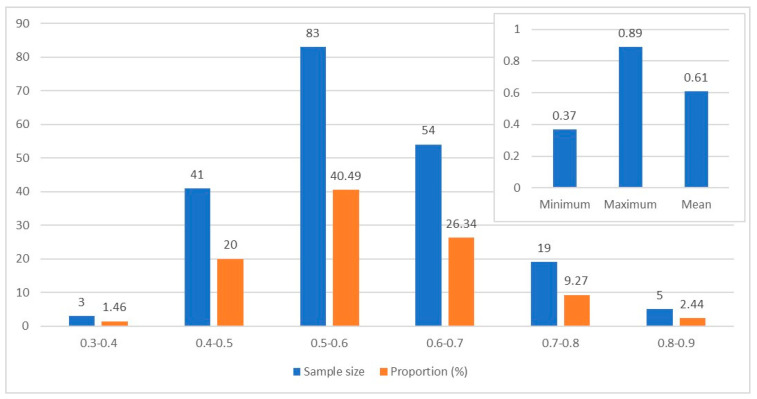
Distribution of the pig farm biosecurity scores in the study area.

To ensure the accuracy of the data used in this paper, reliability and validity tests were conducted on each questionnaire item. Internal consistency, stability, and accuracy were assessed, and the results were promising. The Cronbach coefficients were all greater than 0.7, the KMO test value was 0.6837, and the Bartlett sphericity test was significant at a 1% statistical level. These results suggest that the data used in this paper were highly reliable and valid, allowing for follow-up analysis. The pig farm biosecurity level was determined using the projection pursuit model based on the genetic algorithm, estimated using Matlab R2016. The results of this analysis are presented in Figure 3.

The findings in Figure 3 reveal that the biosecurity levels at the pig farm were generally low, with significant differences observed. Previous studies have suggested that a biosecurity score greater than 0.8 indicates a small risk, while a score greater than 0.6 indicates a medium risk. A score lower than 0.6 indicates a relatively high risk, and a score lower than 0.4 indicates a high risk of biosecurity breach [50]. The sample farmers had an average biosecurity score of 0.61, indicating a medium-level risk. However, this score was considered relatively fragile and at risk of decreasing. The range of scores varied from a minimum of 0.37 to a maximum of 0.89, indicating a significant difference in biosecurity levels among the farmers. The distribution of scores showed that 40.49% of samples scored between 0.5 and 0.6, while 26.34% scored between 0.6 and 0.7. This means that 66.83% of the sample farmers had medium to low biosecurity levels, indicating a potential risk for the expansion of biosecurity risks at any time. Out of all the farms, only 38.05% had a biosafety score greater than 0.6. Among these, only five farms had a score greater than 0.8, which accounted for a mere 2.44%. On the other hand, there were three farms with a score of less than 0.4, accounting for 1.46%. This suggests that there are significant weak links in some aspects of the farmer biosafety system, which has become a bottleneck in the overall effectiveness of the system.

**Figure 4:**
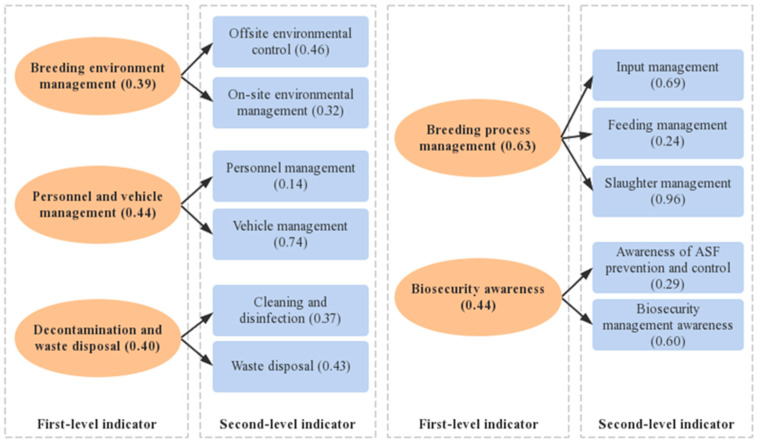
Pig farm biosecurity classifications.

To identify potential weaknesses in the farm’s biosecurity system, a classification analysis was performed on the biosecurity levels. The results, shown in Figure 4, indicate that the first-level indicators were ranked in descending order as follows: breeding process management, biosecurity awareness, personnel and vehicle management, decontamination and waste treatment, and breeding environment management. The study revealed that farmers tend to prioritize breeding process management over breeding environment management. The scores for secondary indicators revealed that slaughter management (0.96) and vehicle management (0.74) were given more importance than other factors, which was in line with the key task of preventing and controlling ASF during production. While most indicators for biosecurity management were satisfactory, scores for personnel management (0.14) and feeding management (0.24) were significantly lower. This highlights major biosecurity shortcomings in these two areas. However, the biosecurity management knowledge score was 0.60, indicating that farmers do recognize the importance of biosecurity management.

### 4.2. The Factors Affecting Farmers’ Biosecurity Level

Prior to estimating the Tobit model using Stata 16.0 software, a multicollinearity test was performed. The results of the test indicated that the variance inflation factor for each variable was below 10, thus indicating an absence of multicollinearity issues. The regression analysis included the farms’ production and operation, breeding environment, and epidemic knowledge variables. Four models (1) to (4) were obtained, and their empirical results are presented in Table 4. The variables’ signs and significance were consistent, indicating a good level of robustness in the model. The model’s goodness of fit improved continuously, as evidenced by the chi-square of the model’s likelihood ratio passing the 1% significance test and the gradual increase in the Pseudo R2 value.

The results of the model (4) estimation indicate that the biosecurity level of farms was significantly affected by their basic characteristics, production and management, breeding environment, and epidemic awareness characteristics. As a result, hypotheses 1 to 4 were verified to varying degrees. The study found that gender had a statistically significant impact on farm biosecurity at a 1% level, with a positive coefficient. Interestingly, this result contradicts theoretical expectations and indicates that male decision makers were more likely to prioritize and improve farm biosecurity measures. The study found that technical training had a statistically significant positive impact on the biosecurity of farms at a 1% level. This result aligns with theoretical expectations and suggests that farmers who received training on biosecurity measures were able to apply this knowledge to improve the biosecurity of their farms. The study found that both the size of the farm and the proportion of farm income had a significant positive impact on the level of biosecurity implemented by farmers. This result was consistent with theoretical expectations and suggests that farmers with larger farms and a higher proportion of income from farming are more likely to take proactive measures to improve biosecurity when faced with the risk of foreign diseases.

According to the study, both production organization and government inspections had a significant positive impact on the biosecurity levels of farms, with a statistical significance of 1%. The results were consistent with theoretical expectations. Farmers who were part of relevant production organizations or received more policy inspections had higher levels of biosecurity. The knowledge of ASF symptoms and mandatory culling policies had a significant and positive impact on the biosecurity of farms at both 1% and 5% statistical levels. This finding is consistent with theoretical expectations and indicates that farmers who possess more knowledge about ASF and its associated policies tend to have higher levels of biosecurity.

## 5. Discussion

### 5.1. Farmers’ Biosecurity Level

Research demonstrates the crucial significance of biosecurity in pig farms. Its implementation not only enhances the resilience of pig farms but also prevents the introduction and spread of new pathogens, including respiratory and gastrointestinal diseases. It helps identify vulnerabilities in production, enhance production methods, increase farm productivity, and ultimately contributes to public health. Furthermore, antibiotics can cause animal-derived products to contaminate farms with drug residues, so farm biosecurity serves as an effective tool for mitigating antibiotics and ensuring animal health, production, and welfare [51]. The assessment of the biosafety of pig farms involves both external and internal biosafety measures [41,52]. The interviews were conducted with pig farms in the western highlands of Cameroon and categorized their biosecurity practices into three areas: isolation, traffic control, and cleaning and disinfection. The authors also highlighted the importance of addressing aspects such as pig housing design in order to improve biosecurity measures. To develop a more comprehensive biosecurity index system, we identified five aspects of biosecurity measures based on related research involving the whole process of pig breeding. This system addressed deficiencies in areas like breeding environment that were not fully considered in previous studies.

Previous studies have utilized descriptive statistics and cluster analyses to assess the biosecurity measures of sampled farmers and identify potential shortcomings. For instance, biosecurity measures commonly implemented in intensive and indoor farming settings were challenging to implement on extensive farms [53]. Additionally, several studies have employed mathematical and statistical approaches to determine the level of biosecurity. The biosecurity of medium and large pig farms was assessed, and it was found that the herds had high external and good internal biosecurity [54]. This study aimed to evaluate biosecurity levels using a mathematical statistics model. The projection pursuit model’s calculation showed that the average biosecurity level was 0.61, indicating that the overall biosecurity was relatively fragile and could easily fall. The study found that there were significant differences in biosecurity measures between farms, with a minimum of 0.37 and a maximum of 0.89. The low biosecurity levels observed in the sample farms were attributed to both internal and external factors. Of all the primary indicators, environmental management had the lowest score. Although farmers pay more attention to the management of the breeding process and environment after the outbreak of African swine fever, many small and medium-sized farmers still have problems with traditional breeding. They are unable to fully comply with the biosafety standards in terms of site selection and regional layout of breeding farms. Additionally, the secondary indicators of personnel management (0.14) and feeding management (0.24) were significantly lower than the other indicators. To ensure biosecurity in personnel management, it is necessary to implement standardized measures such as restricting access to outsiders or returning employees who may have been to places with a potential outbreak risk. Additionally, strict cleaning, disinfection, and isolation procedures should be followed before entering the farm site. However, the majority of farmers usually carry out only basic questioning, cleaning and disinfection, which makes it easier for the virus to be brought into the farm.

### 5.2. The Factors Affecting Farmers’ Biosecurity Level

In this study, it was demonstrated that the awareness of ASF symptoms positively impacted the farms’ biosecurity at 1% statistical levels. However, the farmers who were interviewed lacked personal experience and knowledge in dealing with the epidemic. Additionally, the complexity of ASF and the detection technology made it difficult for ordinary farmers to understand and master [40]. Therefore, there was a lack of understanding of the disease’s consequences and knowledge of prevention and control, as was also noted in a previous study [55]. This lack of comprehension makes it challenging to implement immediate biosecurity measures against ASF, as demonstrated by a study conducted on pig farmers in Hunan Province, China [17].

The biosecurity level of farms was found to be positively correlated with both the scale of the farm and the proportion of income derived from farming. Farmers who rely heavily on breeding as a source of income are less tolerant of income fluctuations caused by epidemic risks. As a result, they are more motivated to enhance their biosecurity measures. Meanwhile, large-scale pig farmers can benefit from economies of scale and lower unit costs for biosecurity prevention and control, which is why they are proactively improving their biosecurity practices [56]. According to the study, farmers who are affiliated with production organizations are more inclined to enhance their biosecurity measures. These organizations facilitate access to advanced knowledge and technology and, through vertical collaboration, encourage farmers to prioritize their biosecurity to safeguard the collective. The effectiveness of controls and prevention measures relies heavily on conscientiousness and timely implementation by those responsible for them [57]. Biosecurity measures are often not strictly followed by farmers due to information asymmetry, difficulties in identifying potential threats, and the high costs associated with implementing such measures [34,58].

The study revealed that technical training had a noteworthy positive impact on the biosecurity of farms. Biosecurity measures are needed for all aspects of the breeding process. The daily tasks involve a combination of physically demanding work and making crucial decisions to prevent and control the spread of diseases. As a result, there is a need to enhance the training and support provided for decision makers in the field of biosecurity. Increased frequency of government inspections raises the likelihood of farmers being found in violation of biosecurity requirements [59]. Thus, increased government involvement has the potential to enhance the awareness of farmers regarding diseases and the associated risks, ultimately leading to improved biosecurity measures [17]. According to the analysis of the determinants of biosecurity behavior among British cattle and sheep farmers, enhancing their understanding of animal disease policy management can lead to better biosecurity practices [10].

Despite China being in a ‘post-ASF’ era, a significant percentage, 36.09%, of farmers still believe that the risk of ASF is large or very large. As a result, promoting appropriate biosecurity practices by strengthening biosecurity awareness, educating pig farmers on ASF, and instructing small-scale producers on responsible pig-raising practices is crucial [31,60]. This is further corroborated by the impact factor analysis. When the farmers were asked about the decisions they would make if biosecurity requirements were more stringent in the future, 85.37% of farmers expressed a willingness to actively seek ways to meet higher standards. This aligns with findings from a study on pig farmers in northern Uganda, indicating a general willingness among farmers to improve their biosecurity practices [8].

The results of this study offer valuable policy insights for enhancing pig farm biosecurity against diseases like ASF. Strategies such as improving the breeding environment and personnel management, intensifying awareness of ASF prevention and control, providing technical training, promoting policy publicity, encouraging farmers to join production organizations, and increasing government supervision can help to improve the overall biosecurity situation on pig farms.

However, this study had some limitations. The development of a biosecurity system is a complex undertaking that requires a comprehensive approach. This study has primarily focused on biosecurity measures from the farmers’ viewpoint without exploring other stakeholders in the industry or government. Future research could explore the broader framework. Additionally, it would be valuable to investigate why some farmers opt to withdraw from breeding, as this could uncover the factors that hinder the improvement of biosecurity measures.

## 6. Conclusions

This study aimed to evaluate the biosecurity level of pig farmers and analyze the factors that affect their biosecurity level. The findings of this study suggest that improving pig farm biosecurity can lead to increased resilience, stable farm incomes, and guarantee pork supply, which in turn can contribute to social stability. Overall, this research highlights the importance of biosecurity measures in the pig farming industry.

The biosecurity levels in pig farms were generally low, as most farmers did not have a systematic high-level biosecurity system in place. This led to weak disease prevention and control, which posed a serious threat to the recovery and improvement of pig production capacity, as well as the sustainable and healthy development of the pig industry. Additionally, serious gaps and weaknesses were found in the biosecurity systems of pig farms, resulting in a short “barrel effect”. Our study revealed that several factors, such as farmer gender, technical training, farm size, income proportion, production organization, government inspections, awareness of ASF symptoms, and knowledge of the compulsory culling policy, significantly impacted the biosecurity level of farms. The majority of farmers displayed a willingness to enhance their biosecurity measures. Thus, increasing awareness of preventive measures and improving epidemiological knowledge can considerably enhance the biosecurity levels of pig farms in China. Further studies should investigate the influence of the government–farmer relationship on biosecurity systems. Further research is required to understand the reasons behind the cessation of pig farming in the post-ASF era, as well as the impact of biosecurity management on their production and operational capabilities.

## Figures and Tables

**Table 1:** Basic information for the interviewed farmers.

Variables	Category	Sample Size	Proportion (%)
Gender	Male	156	76.10
	Female	49	23.90
Education	Primary school and below	78	38.04
	Junior high school	84	40.98
	Senior high school/Technical secondary school	28	13.66
	College and above	15	7.32
Age	under 35	9	4.39
	35–44	36	17.56
	45–54	103	50.24
	55–64	48	23.42
	65 and above	9	4.39
Job	Yes	53	25.85
	No	152	74.15
The proportion of breeding income	25% or less	19	9.27
	25–49%	53	25.85
	50–74%	62	30.25
	75% and above	71	34.63
Breeding scale ^1^	Under 100	139	67.80
	100–499	58	28.29
	500–999	4	1.95
	1000–1999	2	0.98
	2000 and above	2	0.98

^1^ Breeding scale refers to the number of fattening pigs sold on the farm each year, the same below.

**Table 2:** Calculation index system for pig farm biosecurity.

First-Level Indicators	Second-Level Indicators	Third-Level Indicators
Breeding environment management	Offsite environmental control ^1^	More than 500 m from residential areas, trunk lines, other farms, etc.
		Set up greening, fences, and other epidemic prevention isolation belts
		A production area of more than 50 m from other regions
	On-site environmental management ^2^	Field room designed for professionals
		Separation of clean and dirty
		No other animals such as chickens, ducks, dogs, etc.
		Anti-mosquito, rat, bird, and other facilities
Breeding process management	Input management	Regular source of pigs
		Introduced breeding pig isolation area
		Classified storage of feed, medicine, and other inputs
		Feeding without swill
	Feeding management	An all-in, all-out breeding method
		Staffed by a veterinarian or professional technician
		On-site epidemic prevention detection
		Sick pigs and healthy pigs from different breeders
		Sick pig isolation area
		Reasonable dosage of veterinary drugs
		Not fed human medicine
		Breeding file records
	Slaughter management	Sterilization management of pig houses after pig slaughter
		Pigs do not return to the farm after leaving the farm
		Animal supervision agency personnel present when live pigs are released for slaughter
		Quarantine certificates for the live pigs released for slaughter
Personnel and vehicle management	Personnel management	Formal procedures for personnel entering the pig farm
		Reporting procedures before entering the venue for external personnel or personnel returning from vacation
		Background reviews for the recent activities of external personnel or those returning from vacation
		A special isolation room for staff returning to the field
		Personnel unable to visit other livestock, poultry breeding, or trading venues before entering
	Vehicle management	An external pig transporter registered with animal husbandry and veterinary authorities
		Outside vehicles not allowed to directly enter the on-site living area
		External pig transport vehicles not directly counted in the on-site production area
		External feed trucks not allowed to directly enter the on-site production area
		Cleaning, disinfection, and drying measures before unloading
Decontamination and waste treatment	Cleaning and disinfection	Well-established disinfection measures in the field
		Staff ingredients for cleaning and disinfection before entering the venue
		Disinfection items contaminated by sick and dead pigs
		Outsourced meat not allowed
		Cleaning and disinfection of pigs before and after sale
		Various disinfectants to be used
	Waste treatment	Manure is treated by dry and wet (solid–liquid) separation
		Standardized handling of sick and dead pigs
		Normative manure disposal
		Normative sewage treatment method
		Normative disposal of syringes, expired medications, and packaging
Biosecurity awareness	Awareness of ASF prevention	Familiar with the ASFV detection process
		Familiar with the ASFV detection technology
		Government reporting requirements for discovered or suspected ASFV
	Biosecurity management awareness	Familiar with the content or requirements of farm biosecurity management
		Familiar with farm biosecurity technical specifications
		Agreement that the most effective way to prevent and control pig diseases is good biosecurity management
		Funds and human resources to be invested in biosecurity improvements
		Agreement that early warning is more effective than treatment for epidemic prevention and control

^1^ Off-site environmental controls refer to preventing external items, such as personnel, vehicles, and other farms, from carrying imported harmful pathogens into the farm through control. ^2^ On-site environmental control refers to the prevention of cross-infection between internal sick and dead pigs or infected materials and safe pig herds, as well as the spread of pathogens from other biological agents, such as chickens, dogs, etc., raised on the farm, and birds, mosquitoes, mice, etc., outside the farm.

**Table 3:** Variable definitions and descriptive statistics.

Variable Name	Variable Definitions
Explained variable	
Farmers’ biosecurity level	Calculated Score
Explanatory variables	
Farmer characteristics	
Gender	Female = 0; Male = 1
Age	35 and below = 1; 35–44 = 2; 45–54 = 3; 55–64 = 4; 65 and above = 5
Education	Primary school and below = 1; Junior high school = 2; Senior high school/Technical secondary school = 3; College and above = 4
Technical Training	No = 0; Yes = 1
Production and operation characteristics	
Years of farming	5 years and below = 1; 5–9 = 2; 10–14 = 3; 15–19 = 4; 20 and above = 5
Farm size	100 and above = 1; 100–499 = 2; 500–999 = 3; 1000–1999 = 4; 2000 and above = 5
Social position ^1^	No = 0; Yes = 1
Percentage of farm income	25% or less = 1; 25%–49% = 2; 50%–74% = 3; 75% and above = 4
Farming environment characteristics	
Production organization ^2^	No participation = 0; Participation = 1
Farm insurance	No = 0; Yes = 1
Government inspection	1 and below = 0; 1–6 = 1; 7–12 = 2; 12 and above = 3
Epidemic awareness characteristics	
Awareness of ASF symptoms	Know the disease symptoms
Awareness of mandatory culling policy	No = 0; Yes = 1
Willingness to report the epidemic	No = 0; Yes = 1

^1^ Social position refers to whether the farmers are party members, cadres at the village level and above, and family members engaged in the promotion of animal husbandry technology among family members. ^2^ Production organization refers to an economic organization in which individual producers join forces to produce collectively.

**Table 4:** Regression results from factors affecting the pig farmers’ biosecurity level.

	Model (1)	Model (2)	Model (3)	Model (4)
Gender	0.0430 ***	0.0346 ***	0.0350 ***	0.0308 ***
	(0.0131)	(0.0125)	(0.0118)	(0.0110)
Age	−0.0052	−0.0030	−0.0022	−0.0016
	(0.0079)	(0.0077)	(0.0068)	(0.0063)
Education	0.0125	0.0059	0.0053	0.0015
	(0.0088)	(0.0071)	(0.0069)	(0.0063)
Technical Training	0.0652 ***	0.0560 ***	0.0417 ***	0.0323 ***
	(0.0120)	(0.0112)	(0.0108)	(0.0105)
Breeding years		0.0004	0.0006	−0.0003
		(0.0042)	(0.0037)	(0.0034)
Breeding scale		0.0542 ***	0.0430 ***	0.0369 ***
		(0.0090)	(0.0092)	(0.0075)
Job		0.0043	−0.0039	−0.0030
		(0.0128)	(0.0117)	(0.0107)
The proportion of breeding income		0.0174 ***	0.0175 ***	0.0160 ***
		(0.0050)	(0.0050	(0.0048)
Production organization			0.0793 ***	0.0728 ***
			(0.0225)	(0.0202)
Breeding insurance			−0.0041	−0.0025
			(0.0118)	(0.0113)
Government inspection			0.0222 **	0.0215 ***
			(0.0085)	(0.0080)
ASF symptoms awareness				0.0109 ***
				(0.0026)
Mandatory culling policy awareness				0.0243 **
				(0.0100)
Willingness to report the epidemic				0.0116
				(0.0159)
Constant	0.4810 ***	0.4003 ***	0.4001 ***	0.3790 ***
	(0.0359)	(0.0385)	(0.0381)	(0.0376)
Region	Controlled	Controlled	Controlled	Controlled
Log pseudolikelihood	212.9039	234.6992	251.8200	266.8606
Prob > chi2	0.0000	0.0000	0.0000	0.0000
Pseudo R2	−0.1225	−0.2374	−0.3277	−0.4070
N	205	205	205	205

Notes: ** and *** are statistically significant at the level of 10%, 5%, and 1%, respectively; standard error is given in parentheses.

## Data Availability

The data that support the findings of this study are available from the corresponding author upon reasonable request.
